# Vitamin D Alleviates Anxiety and Depression in Elderly People with Prediabetes: A Randomized Controlled Study

**DOI:** 10.3390/metabo12100884

**Published:** 2022-09-20

**Authors:** Evangelia Zaromytidou, Theocharis Koufakis, Georgios Dimakopoulos, Despina Drivakou, Stavroula Konstantinidou, Pantelitsa Rakitzi, Maria Grammatiki, Eleni Manthou, Athanasios Notopoulos, Ioannis Iakovou, Anna Gotzamani-Psarrakou, Kalliopi Kotsa

**Affiliations:** 1 Nuclear Medicine Department, Hippokration General Hospital, 54642 Thessaloniki, Greece; 2 Division of Endocrinology and Metabolism, First Department of Internal Medicine, AHEPA University Hospital, Medical School, Aristotle University of Thessaloniki, 54636 Thessaloniki, Greece; 3 BIOSTATS, Epirus Science and Technology Park Campus, University of Ioannina, 45500 Ioannina, Greece; 4 Theageneio Hospital, 54639 Thessaloniki, Greece; 5 Second Academic Nuclear Medicine Department, Academic General Hospital of Thessaloniki “AHEPA”, Aristotle University of Thessaloniki, 54636 Thessaloniki, Greece; 6 Medical School, Aristotle University of Thessaloniki, 54124 Thessaloniki, Greece

**Keywords:** vitamin D, prediabetes, anxiety, depression, elderly

## Abstract

Older people are prone to frailness, present poor adherence to pharmacotherapy, and often have adverse drug effects. Therefore, it is important to develop effective and safe interventions to mitigate the burden of anxiety and depression disorders in this population. The aim of this study was to investigate the effect of vitamin D supplementation on the anxiety and depression status of elderly people with prediabetes. Participants were randomly assigned a weekly dose of vitamin D_3_ of 25,000 IU (*n* = 45, mean age 73.10 ± 7.16 years) or nothing (*n* = 45, mean age 74.03 ± 7.64 years), in addition to suggested lifestyle measures. The State-Trait Anxiety Inventory subscales (STAI-T and STAI-S) and the Patient Health Questionnaire-9 (PHQ-9) were used to evaluate anxiety and depression levels, respectively, at baseline, 6, and 12 months. A total of 92.68% of the participants in the vitamin D group and 97.14% of the controls exhibited vitamin D deficiency (<20 ng/mL) at baseline. Mean STAI-T scores were lower in supplemented individuals than in the control group at 6 (38.02 ± 9.03 vs. 43.91 ± 7.18, *p* = 0.003) and 12 months (32.35 ± 7.77 vs. 44.97 ± 7.78, *p* < 0.001). The same pattern was evident for STAI-S scores at 6 (37.11 ± 7.88 vs. 43.20 ± 9.33, *p* = 0.003) and 12 months (32.59 ± 6.45 vs. 44.60 ± 9.53, *p* < 0.001). Supplemented participants demonstrated lower mean PHQ-9 scores compared to controls at 6 (15.69 ± 6.15 vs. 19.77 ± 8.96, *p* = 0.021) and 12 months (13.52 ± 5.01 vs. 20.20 ± 8.67, *p* < 0.001). Participants with deficiency and insufficiency at baseline experienced equal benefits of supplementation in terms of anxiety and depression scores. In conclusion, in a high-risk population, a weekly vitamin D supplementation scheme was effective in alleviating anxiety and depression symptoms. More studies are needed to elucidate the relevant mechanisms.

## 1. Introduction

Older age has been identified as a major risk factor for vitamin D deficiency related to multiple causes, including reduced vitamin D dietary intake, increased adiposity, decreased vitamin D cutaneous synthesis, and altered physical activity leading to limited exposure to sunlight [1]. Among older people, vitamin D deficiency has been associated with higher mortality odds [2], while restoration of adequate concentrations appears to improve neuromuscular function and reduce the risk of falls in this population [3]. A wealth of epidemiological data suggests an association between vitamin D deficiency and several disorders whose prevalence increases with age, including cancers, as well as cardiovascular, mental, and metabolic diseases; however, to date, there is no robust evidence to support the role of universal supplementation in reducing these risks [4].

Prediabetes represents an important public health concern, not only because it significantly increases the risk of progression to diabetes, but also because it has been associated with the development of diabetes-related complications [5]. Previous research suggests a bidirectional relationship between impaired glucose metabolism and depression, which means that each condition can increase the risk of developing the other [6]. Furthermore, anxiety has been shown to accelerate the progression of prediabetes to type 2 diabetes (T2D) [7]. Although the link between prediabetes and psychiatric disorders is obviously mediated by complex factors, such as the burden of comorbidities (e.g., cardiovascular disease and physical disabilities), the association appears to remain significant, even after adjustment for potential confounders, implying the existence of common pathophysiological pathways, such as systemic inflammation, between the two entities [8].

The available data suggest that the coexistence of diabetes and depression has a negative impact on the quality of glycemic control by affecting the patient’s ability to self-manage and comply with the therapeutic plan [9]. In addition, older people are prone to frailness, present poor adherence to pharmacotherapy, and often experience adverse drug effects [10]. Therefore, it is important to develop effective and safe interventions to mitigate the burden of anxiety and depression disorders in this population. Furthermore, while the association between dysglycemia and mood disorders on the one hand, and aging and depression on the other hand has been extensively described in the literature, little attention has been paid to the simultaneous existence of the three conditions (old age, prediabetes, and mood disorders) that are believed to largely interact with each other. This article reports on the effects of vitamin D supplementation on the anxiety and depression status of Greek elderly people with prediabetes over a period of 12 months.

## 2. Materials and Methods

### 2.1. Study Population

This is a sub-study of the trial “The Effect of vitamin D on People with Prediabetes,” whose primary outcome was to investigate the potential benefits of vitamin D supplementation on glycemic markers of elderly individuals with prediabetes. Secondary outcomes included the effects of vitamin D on anxiety and depression status, as well as on osteoporosis markers of the study population. Details on the design and research methods of this study have previously been reported [11].

In summary, the participants were men and women over 60 years old who were diagnosed with prediabetes, according to the criteria of the American Diabetes Association [12]. Exclusion criteria included a history of diabetes, any medical conditions that could affect study results or increase the risk of complications after vitamin D supplementation (indicatively: nephrolithiasis, hypercalcemia, hyperparathyroidism, sarcoidosis, and chronic renal disease/stages 3 to 5), malignancies, chronic inflammatory or rheumatic diseases, and psychiatric conditions, including mood or anxiety disorders that require pharmacotherapy.

### 2.2. Intervention

Among the 381 individuals initially screened, 105 were diagnosed with prediabetes, and 90 eventually met the inclusion criteria and agreed to participate. Using an open-label design, they were randomly assigned by computer code to receive a weekly dose of vitamin D_3_ of 25,000 IU in the form of an oral solution administered once a week (intervention group; *n* = 45) or nothing (control group; *n* = 45). Both groups were advised to adopt specific lifestyle changes, according to current recommendations for the prevention of diabetes, that is, at least 150 min per week of moderate intensity aerobic activity, and target a weight loss of 7% within 3 months following the Mediterranean diet [13]. Participants were seen monthly for the first 3 months of the follow-up period, and subsequently, every 3 months until the end of the study. Each visit included a consultation with a physician and a dietitian to resolve potential problems, while subjects were contacted by telephone monthly by members of the research team to ensure adherence to the diet and physical activity plans and compliance with supplement use. The latter was also determined from the number of empty medication boxes returned at each visit.

### 2.3. Assessment of Anxiety and Depression Status

The state of anxiety was evaluated with the Spielberger Inventory of State-Trait Anxiety Self-Esteem Scale (STAI)-Form Y. This 40-item self-report questionnaire form is an adult stress psychometric assessment tool that has been validated in the Greek population [14]. Participants indicate the degree of agreement or disagreement on each issue (not at all, somewhat, moderately, too much). The test is intended for people over 10 years of age and, after completing the questionnaire, it results as a four-scaled anxiety index. The first 20 questions reflect the psychological and physiological transient reactions directly related to adverse situations at a specific time (state anxiety/STAI-S). The next 20 questions refer to a personality trait, describing individual differences related to a tendency to present state anxiety (trait anxiety/STAI-T). The completion duration ranges from 10–20 min, it is reproducible, and the reliability of repetitive measurements is considered satisfactory. The range of possible scores for the STAI-Form Y varies from a minimum score of 20 to a maximum score of 80 on both the STAI-T and STAI-S subscales. STAI scores are commonly classified as “no or low anxiety” (20–37), “moderate anxiety,” (38–44) and “high anxiety” (45–80) [15].

Depression levels were evaluated with the Patient Health Questionnaire-Depression (PHQ-9). PHQ-9 is the nine-item depression subscale of the Patient Health Questionnaire and is a widely used tool to help primary care physicians diagnose depression and monitor treatment [16]. PHQ-9 has been validated in the Greek population [17] and is suitable for use with adults, evaluating the presence of symptoms in the previous two weeks. Possible scores range from 0 to 27, where higher scores indicate more intense depressive symptoms. A PHQ-9 score of more than 10 has a sensitivity of 88% and a specificity of 88% for major depression [18]. Severity categories are as follows: Mild depression—5 to 9, moderate depression—10 to 14, moderately severe depression—15 to 19, and severe depression—20 to 27. All evaluations were performed in both groups at baseline, 6, and 12 months.

### 2.4. Ethical Considerations

All procedures followed were in accordance with the ethical standards of the responsible Committee on Human Experimentation (institutional and national) and with the Declaration of Helsinki of 1975, as revised in 2008. Informed consent was obtained from all study participants. The research protocol was approved by the Ethics Committee of the Aristotle University of Thessaloniki (approval number: 260/19-04-16). The trial was retrospectively registered on the ISRCTN registry for clinical trials (https://www.isrctn.com/ISRCTN51643592 accessed on 18 August 2022), with the registration number ISRCTN51643592.

### 2.5. Statistical Methods

Means and standard deviations were used to describe the scores of the main outcomes throughout the 12-month follow-up period. General linear models for repeated measures were applied to assess statistically significant differences in all cases, and the Bonferroni adjustment was applied for multiple comparison tests. Mixed models were applied for the intention-to-treat analysis for the levels of the outcomes. Statistical significance was set at 0.05, and the analysis was performed using SPSS v26.0 software.

## 3. Results

### 3.1. Study Population and Laboratory Markers

Among the 45 individuals randomized in each group, 42 completed the trial in the vitamin D group and 35 in the control group. All dropouts were due to personal reasons, and no adverse effects of vitamin D administration were observed. Participants in the intervention and control group presented comparable baseline characteristics in terms of age (73.10 ± 7.16 vs. 74.03 ± 7.64 years, respectively), anthropometric characteristics, and glycemic markers. Table 1 shows the baseline demographic, laboratory, and anthropometric characteristics of the two groups, in detail. Among the participants who completed the trial, 29/42 (69.05%) in the vitamin D group and 22/35 (62.86%) in the control group (*p* = 0.56) had impaired fasting glucose (IFG) (fasting plasma glucose: 100–125 mg/dL) at baseline. A total of 16/42 (38.10%) in the supplementation group and 17/35 (48.57%) in the control group (*p* = 0.35) had impaired glucose tolerance (IGT) (2-h plasma glucose in 75 g oral glucose tolerance: 140–199 mg/dL) at baseline. A total of 11/42 (15.28%) in the vitamin D group and 12/35 (34.29%) in the control group had combined IFG and IGT at baseline. Finally, all participants in both groups had glycated hemoglobin levels between 5.7 and 6.4% at baseline.

Regarding vitamin D status, the percentage of 25(OH)D-deficient individuals in the intervention group decreased from 92.86% at baseline, to 69.05%, 57.14%, and 52.38% at 3, 6, and 12 months, respectively (*p* < 0.001 for all comparisons). The respective percentages in the control group were 97.14% (baseline), 97.14% (3 months), 91.43% (6 months), and 82.86% (12 months), and the differences between all time points were not significant. Controls did not experience significant changes during the follow-up period, starting from baseline values equal to 19.85 ± 5.72 ng/mL and reaching 19.94 ± 5.82, 19.68 ± 5.96, and 20.30 ± 7.08 ng/mL at 3, 6, and 12 months, respectively. The supplemented participants had baseline 25(OH)D values equal to 19.98 ± 6.73 ng/mL and showed a significant increase in vitamin D levels at 3 (23.56 ± 7.81 ng/mL, *p* < 0.001), 6 (26.56 ± 8.64 ng/mL, *p* < 0.001), and 12 months (28.71 ± 9.03 ng/mL, *p* < 0.001) compared to baseline. In terms of differences between the 2 groups, the supplemented participants presented higher concentrations compared to the controls, at 6 (26.56 ± 8.64 vs. 19.68 ± 5.96 ng/mL, respectively, *p* = 0.002), and 12 months (28.71 ± 9.03 vs. 20.30 ± 7.08 ng/mL, respectively, *p* < 0.001). 

Regarding inflammation markers and, more specifically, white blood cells (WBC), the vitamin D group had levels comparable to the control group at baseline (6.24 ± 1.52 vs. 6.17 ± 1.69 × 10^3^, *p* = 0.84) and 12 months after the start of supplementation (6.46 ± 1.47 vs. 6.47 ± 1.70 × 10^3^, *p* = 0.97). However, controls experienced a significant increase in WBC levels at 12 months compared to baseline (*p* < 0.001), an observation that was not replicated in vitamin D-supplemented participants. Regarding anthropometry, the supplemented participants demonstrated a significant reduction in BMI at 12 months compared to baseline (29.50 ± 4.10 vs. 29.90 ± 4.16 kg/m^2^, *p* = 0.047). In contrast, no differences were observed in body weight and waist circumference (WC) during the follow-up period: 74.86 ± 12.97 (baseline) vs. 74.97 ± 12.64 (12 months) kg, *p* = 0.74, and 97.17 ± 13.46 (baseline) vs. 97.36 ± 13.42 (12 months) cm, *p* = 0.58, respectively. Controls did not show significant differences in anthropometric markers at 12 months compared to baseline (BMI: 30.46 ± 4.63 vs. 30.29 ± 4.14 kg/m^2^, *p* = 0.41; body weight: 77.39 ± 13.71 vs. 77.22 ± 12.96 kg, *p* = 0.62; WC: 97.54 ± 11.48 vs. 97.91 ± 11.26 cm, *p* = 0.64). The effects of supplementation on the glycemic markers of the participants have been controlled for changes in anthropometry.

### 3.2. Effect of Vitamin D on Anxiety

**Figure 1:**
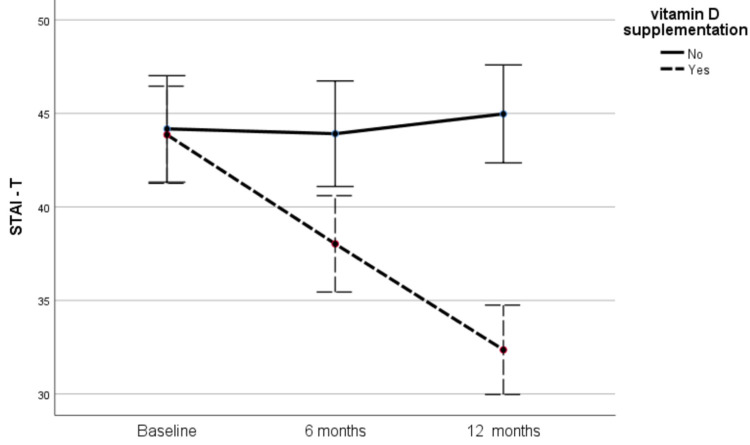
Changes in STAI-T scores in the two groups during the study.

At baseline, the mean value of the STAI-T score for controls and supplemented individuals was 44.17 ± 5.94 and 43.85 ± 9.40, respectively, and the difference between the two groups was not significant (*p* = 0.87). After 6 and 12 months, the STAI-T values for the supplemented participants decreased significantly to 38.02 ± 9.03 (*p* < 0.001 compared to baseline) and 32.35 ± 7.77 (*p* < 0.001 compared to baseline and 6 months), respectively. The respective values for the control group were 43.91 ± 7.18 and 44.97 ± 7.78, which did not differ significantly from the baseline scores. Table 2 and Figure 1 present the evolution of STAI-T values in the two groups throughout the study period. As shown in Table 2, at 6 and 12 months, supplemented participants had significantly lower STAI-T score values compared to non-supplemented controls (*p* = 0.003 and <0.001, respectively).

**Figure 2:**
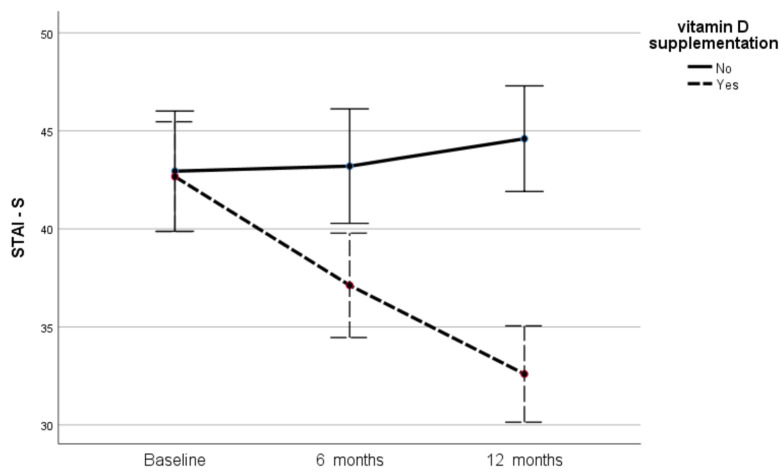
Changes in STAI-S scores in the two groups during the study.

At baseline, the mean value of the STAI-S score for controls and supplemented individuals was 42.94 ± 9.14 and 42.66 ± 8.43, respectively, and the difference between the two groups was not significant (*p* = 0.89). After 6 and 12 months, the STAI-S values for the supplemented participants decreased significantly to 37.11 ± 7.88 (*p* < 0.001 compared to baseline) and 32.59 ± 6.45 (*p* < 0.001 compared to baseline and 6 months), respectively. The respective values for the control group were 43.20 ± 9.33 and 44.60 ± 9.53, with the increase from 6 to 12 months being marginally significant (*p* = 0.043). Table 3 and Figure 2 present the evolution of STAI-S values in the two groups throughout the study. As shown in Table 3, at both 6 and 12 months, the supplemented participants presented significantly lower values of the STAI-S score compared to the controls (*p* = 0.003 and <0.001, respectively).

### 3.3. Effect of Vitamin D on Depression

**Figure 3:**
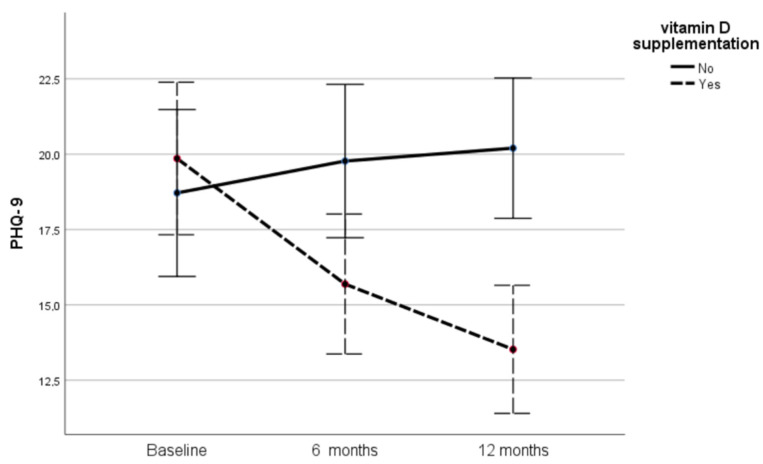
Changes in PHQ-9 scores in the two groups during the study.

Before starting vitamin D supplementation, the two groups had comparable PHQ-9 scores (intervention group: 19.85 ± 7.37 vs. controls: 18.71 ± 9.01, *p* = 0.54). At 6 and 12 months, the score values decreased significantly in the supplemented individuals (15.69 ± 6.15 and 13.52 ± 5.01, respectively, *p* < 0.001 for comparisons between all time-points), while they increased non-significantly in controls (19.77 ± 8.96 and 20.20 ± 8.67, respectively). Table 4 and Figure 3 present the evolution of the PHQ-9 values in the two groups throughout the study period. As shown in Table 4, supplemented participants had significantly lower PHQ-9 score values compared to controls 6 and 12 months after starting vitamin D supplementation (*p* = 0.021 and <0.001, respectively).

### 3.4. Analysis According to 25(OH)D Status

According to the guidelines of the Endocrine Society for vitamin D, deficiency is defined as 25(OH)D levels below 20 ng/mL, while insufficiency is defined as 25(OH)D concentrations between 21 and 29 ng/mL [19]. To explore whether deficient and insufficient individuals experienced equal benefits in terms of mood and anxiety status from supplementation, we performed a separate analysis comparing the scores of supplemented participants who had baseline 25(OH)D concentrations lower than 20 ng/mL to the scores of those who started the trial with levels greater than 20 ng/mL. The analysis produced nonsignificant results (*p* = 0.32 for STAI-T, *p* = 0.23 for STAI-S, and *p* = 0.12 for PHQ-9 at 12 months), suggesting similar benefits in the two groups.

### 3.5. Intention-to-Treat Analysis

As 13 of the 90 randomized participants dropped out of the study (10 from the control group), an intention-to-treat (ITT) analysis was performed to examine the effect of their dropout on the findings. In general, the pattern of the results was not significantly altered. Regarding STAI-T scores, all changes in the intervention group remained significant throughout the follow-up period, although in some cases, the statistical significance decreased compared to the original analysis (baseline vs. 6 months: *p* = 0.007; baseline vs. 12 months: *p* < 0.001; 6 months vs. 12 months: *p* = 0.01). Regarding STAI-S scores, the decrease in the supplemented group remained significant at 6 (*p* = 0.01) and 12 (*p* < 0.001) months compared to baseline values. However, in the ITT analysis, the difference between 6 and 12 months was not significant (*p* = 0.14). Finally, in terms of PHQ-9 scores, the decrease in the vitamin D group remained significant at 12 months compared to baseline (*p* = 0.007), but significance disappeared with respect to differences between baseline and 6 months (*p* = 0.26) and between 6 and 12 months (*p* = 0.78).

## 4. Discussion

In the general elderly population, the benefit of vitamin D supplementation has been primarily related to skeletal outcomes, such as reduced incidence of falls (vitamin D alone) and fractures (vitamin D combined with calcium) [20]. While some studies raised concerns about the safety of high-dose (>100,000 IU), intermittent schemes with respect to the risk of falls [21], others showed similar efficacy and safety profiles between daily, weekly, or monthly administration [22]. However, the effects of supplementation on extraskeletal outcomes, and particularly those related to mental health in the elderly, have not been adequately studied. To our knowledge, this is the first study to examine the potential role of vitamin D in alleviating anxiety and depression symptoms in elderly people with prediabetes. Our findings suggest an improvement in both entities at 6 and 12 months after starting supplementation, accompanied by an excellent safety profile. Although the recommended dose of vitamin D for adults older than 70 years is 800 IU/day [19], in this trial, we used a higher mean daily dose. This is because previous studies have shown that supplementation should target serum 25(OH)D concentrations higher than those known to be optimal for skeletal health to show an improvement in extraskeletal outcomes [11]. The natural history of prediabetes in the elderly might be different from that of younger individuals. As identified in the study by Rooney et al. in which 3412 older adults were recruited [23], the prevalence of prediabetes in this population is high (ranging from 29 to 73%, according to the diagnostic criterion used). However, death or return to normoglycemia was more frequent than progression to diabetes. These findings suggest that prediabetes in the elderly may not be a strong diagnostic entity for predicting the future development of diabetes. 

Emerging evidence demonstrates an inverse correlation between plasma vitamin D levels and the risk of clinical depression. A meta-analysis that included data from 31,424 participants showed an increased odds ratio (OR) of depression for the lowest vs. the highest vitamin D concentrations (OR 1.31; 95% confidence interval 1.00–1.71) [24]. However, it is challenging to translate these findings into clinical practice because most relevant studies have a cross-sectional or observational character and are therefore unable to establish causation [25]. For example, people with depression are less likely to participate in outdoor activities compared to healthy controls; therefore, the possibility of reverse causality should be taken into account when trying to interpret these findings. Previous randomized controlled trials (RCT) that enrolled elderly patients with depression provided encouraging results by showing that vitamin D supplementation can improve depression scores in a short period of time (8 weeks) [26]. Furthermore, among women of reproductive age with prediabetes and hypovitaminosis D, co-supplementation with vitamin D and omega-3 improved anxiety, depression, and sleep quality [27].

Similarly, a meta-analysis that included 25 trials with a total of 7534 patients showed that vitamin D is effective in reducing negative emotions (anxiety and depression) [28]. However, a sub-analysis of the data revealed that the effect was particularly evident when supplementation lasted more than 8 weeks and used doses greater than 4000 IU. These findings highlight that inconsistency in the results of various trials could be related to significant heterogeneity in terms of study design, including supplementation dose, baseline 25(OH)D values, intervention period, vitamin D form used for supplementation, sample size, tools used to diagnose anxiety or depression, and population characteristics [4]. In this context, two recent RCTs that examined a potential benefit of vitamin D on mood-related outcomes among healthy individuals produced negative results [29,30]. Hence, it is reasonable to conclude that populations at high risk for mood disorders are expected to benefit more from vitamin D. Furthermore, we observed similar improvements in depression and anxiety scores in individuals who were deficient at baseline compared to those with insufficient status, suggesting that the benefits were consistent across the entire spectrum of 25(OH)D levels. 

The exact mechanisms through which vitamin D can improve anxiety and depression symptoms remain poorly understood. A study involving women with T2D established a positive effect of vitamin D supplementation on mood status, accompanied by a decrease in C-reactive protein levels, implying that down-regulation of systemic inflammation by vitamin D could play a key role [31]. In support of this perspective, we demonstrated a significant increase in WBC only in non-supplemented participants during the study period, suggesting that vitamin D has the ability to suppress inflammatory markers. Animal studies highlight the potential of vitamin D to reduce oxidative stress, decrease elevated levels of neuronal calcium (important drivers of depression), and improve signaling between brain cells [32]. Furthermore, it has been shown that in the presence of mutations in the vitamin D receptor (VDR) gene that alter the binding capacity of vitamin D to VDR and consequently, the formation of calcitriol, the regulation of neurotropic factor synthesis, such as neurotrophins, is impaired [33]. Basic and clinical evidence suggests that depression is associated with structural and neurochemical changes that include altered neurotrophin levels [34]. Thus, vitamin D deficiency could lead to the inability to maintain the equilibrium of these important molecules for brain function and ultimately, to the development of cerebral dysfunction [35]. 

The strengths of our study include its randomized nature, long-term follow-up, the lack of a history of severe mood and anxiety disorders among participants, as well as the ethnically homogeneous study population, taking into account the racial disparities in response to vitamin D supplementation [36]. However, its findings should be interpreted in light of some limitations. Diet, sunlight exposure, physical activity, seasonal fluctuation of 25(OH)D concentrations, and other confounders that affect vitamin D, and possibly mood status, were not taken into consideration; however, randomization and the fact that dietary and exercise instructions were common in the two groups, and the fact that participants were closely monitored to ensure adherence to diet and physical activity plans, should have minimized the effect of these parameters. Furthermore, self-reporting of anxiety and depression symptoms has previously been shown to lead to under-reporting [37]. 

## 5. Conclusions

Previous studies have shown that prediabetes and older age constitute risk factors for the development of anxiety and depression disorders [38,39,40]. Therefore, in this trial we showed that in a high-risk population, a weekly vitamin D supplementation scheme was effective in reducing anxiety and depression levels, while the benefits were similar in those with baseline concentrations in the zone of deficiency and insufficiency. More studies are needed to elucidate the relevant mechanisms and practical aspects of this therapeutic approach, including the profile of subjects who are most likely to benefit and the ideal dose and duration of administration.

## Figures and Tables

**Table 1:** Baseline demographic, laboratory, and anthropometric features of the two groups.

Parameter	Vitamin D Supplementation	Mean	Std. Deviation	*p*-Value
Age (years)	No	74.03	7.63	0.582
	Yes	73.10	7.16	
Males:Females	No	10:35	-	0.796
	Yes	9:36	-	
25(OH)D (ng/mL)	No	19.85	5.72	0.931
	Yes	19.98	6.73	
Fasting glucose (mg/dL)	No	100.09	10.56	0.407
	Yes	98.71	9.40	
Glycated hemoglobin (%)	No	5.87	0.22	0.992
	Yes	5.87	0.21	
Fasting insulin (μΙU/mL)	No	12.71	13.46	0.819
	Yes	12.11	9.66	
Weight (kg)	No	77.22	12.96	0.428
	Yes	74.86	12.97	
Height (cm)	No	159.39	6.09	0.617
	Yes	158.58	7.69	
Waist circumference (cm)	No	97.91	11.26	0.795
	Yes	97.17	13.46	
Total body fat (%)	No	35.00	7.60	0.463
	Yes	36.00	6.30	
Muscle mass (kg)	No	46.69	9.12	0.240
	Yes	44.24	8.99	
Body Mass Index (kg/m^2^)	No	30.29	4.14	0.687
	Yes	29.90	4.16	
Visceral fat (kg)	No	12.77	4.19	0.399
	Yes	11.90	4.68	

**Table 2:** Evolution of STAI-T scores and differences between the two groups throughout the study period.

STAI-T	Vitamin D Supplementation	Mean	Std. Deviation	95% Confidence Interval	
				Lower Bound	Upper Bound
Baseline	No	44.17	5.94	41.32	47.01
	Yes	43.85	9.40	41.25	46.45
6 months	No	43.91	7.18	41.09	46.73
	Yes	38.02	9.03	35.44	40.60
12 months	No	44.97	7.78	42.35	47.59
	Yes	32.35	7.77	29.96	34.74
		**Mean difference (No-Yes)**	**Sig.**	**Lower** **Bound**	**Upper** **Bound**
Baseline		0.31	0.87	−3.54	4.16
6 months		5.89	0.003	2.06	9.71
12 months		12.61	0.000	9.06	16.15

Post hoc pairwise comparisons using the Bonferroni correction, after general linear models for repeated measures were used to compare the STAI-T scores between different time points.

**Table 3:** Evolution of STAI-S scores and differences between the two groups throughout the study period.

STAI-S	Vitamin D Supplementation	Mean	Std. Deviation	95% Confidence Interval	
				Lower Bound	Upper Bound
baseline	No	42.94	9.14	39.87	46.01
	Yes	42.66	8.43	39.86	45.46
6 months	No	43.20	9.33	40.28	46.11
	Yes	37.11	7.88	34.45	39.78
12 months	No	44.60	9.53	41.90	47.29
	Yes	32.59	6.45	30.13	35.05
		**Mean difference** **(No-Yes)**	**Sig.**	**Lower** **Bound**	**Upper** **Bound**
baseline		0.27	0.89	−3.87	4.42
6 months		6.08	0.003	2.12	10.03
12 months		12.00	0.000	8.35	15.65

Post hoc pairwise comparisons using the Bonferroni correction, after general linear models for repeated measures were used to compare the STAI-S scores between different time points.

**Table 4:** Evolution of PHQ-9 scores and differences between the two groups throughout the study period.

PHQ-9	Vitamin D Supplementation	Mean	Std. Deviation	95% Confidence Interval	
				Lower Bound	Upper Bound
Baseline	No	18.71	9.01	15.94	21.48
	Yes	19.85	7.37	17.32	22.38
6 months	No	19.77	8.96	17.22	22.31
	Yes	15.69	6.15	13.36	18.01
12 months	No	20.20	8.67	17.87	22.52
	Yes	13.52	5.01	11.39	15.65
		**Mean Difference (No-Yes)**	**Sig.**	**Lower** **Bound**	**Upper** **Bound**
Baseline		−1.14	0.54	−4.89	2.60
6 months		4.08	0.021	0.63	7.52
12 months		6.67	0.000	3.52	9.83

Post hoc pairwise comparisons using the Bonferroni correction, after general linear models for repeated measures were used to compare the PHQ-9 scores between different time points.

## Data Availability

The data presented in the study are available on request from the corresponding author.

## Trial Registration

The trial was retrospectively registered on the ISRCTN registry for clinical trials (https://www.isrctn.com/ISRCTN51643592 (accessed on 18 August 2022)) with the registration number ISRCTN51643592.
